# Preoperative prediction of aggressive endometrial cancer using multiparametric MRI-based deep transfer learning models

**DOI:** 10.3389/fonc.2025.1694223

**Published:** 2025-11-18

**Authors:** Ran Guo, Ruchen Peng, Yancui Li, Xiuzhi Shen, Jiali Zhong, Ruiqiang Xin

**Affiliations:** Department of Radiology, Beijing Luhe Hospital, Capital Medical University, Beijing, China

**Keywords:** endometrial carcinoma, aggressive, histological grade, deep learning, artificial intelligence, multiparametric magnetic resonance imaging

## Abstract

**Background:**

Accurate preoperative prediction of endometrial cancer (EC) aggressiveness is critical for individualized treatment planning. This study proposes a method for integrating multimodal data to improve the prediction of aggressiveness in EC.

**Methods:**

A total of 207 patients with pathologically confirmed EC were retrospectively enrolled. The patients were randomized (7:3) into a training cohort (n=144) and a test cohort (n=63). All patients underwent preoperative MRI including T2-weighted imaging, diffusion-weighted imaging, apparent diffusion coefficient mapping, and contrast-enhanced T1-weighted imaging (CE-T1WI). Deep learning (DL) models using ResNet50, ResNet101, DenseNet121 were employed to extract deep transfer learning (DTL) features. Three decision-level fusion strategies (mean, maximum, and minimum) were applied to integrate the multi-sequence model outputs, from which the optimal DTL model was selected. Subsequently, a combined clinical-DTL model was constructed by incorporating independent clinical predictors identified through univariate and multivariate logistic regression analyses. Model performance was evaluated using the area under the receiver operating characteristic curve (AUC), clinical utility by decision curve analysis, and goodness-of-fit through calibration curves.

**Results:**

The mean fusion model integrating features from T2WI, ADC, and CE-T1WI (excluding DWI due to suboptimal performance) yielded the best predictive efficacy, with an AUC of 0.963 [95% confidence interval (CI): 0.933–0.992] in the training cohort and 0.925 (95% CI: 0.859–0.990) in the test cohort. The combined clinical-DTL model further achieved AUCs of 0.972 (95%CI: 0.948–0.997) and 0.950 (95%CI: 0.891–1.000) in the training and test cohorts, respectively. Decision curve analysis and calibration analyses confirmed its clinical utility and good model fit.

**Conclusion:**

The proposed DTL model based on multiparametric MRI demonstrates strong performance in preoperatively predicting aggressive EC. The integration of clinical features further enhances model performance, offering a non-invasive tool to support personalized treatment strategies.

## Introduction

Endometrial carcinoma (EC) has become one of the most common gynecologic malignancies in developed countries (1, 2). In recent years, rising global obesity rates and population aging have contributed to a significant increase in its incidence and mortality (3). The 2023 FIGO staging system introduces key updates by incorporating histological type, tumor growth pattern, and molecular classification—factors that critically influence prognosis and guide personalized treatment strategies (4, 5). Histologically, EC is now categorized into two major groups: Non-aggressive tumors and aggressive tumors (5). Any aggressive histology without myometrial invasion is classified as stage IC (previously IA). Aggressive tumors with myometrial involvement are now stage IIC (formerly IB). For early-stage, low-grade EC, lymphadenectomy does not improve progression-free or overall survival but increases surgical complications (e.g., bleeding, infection, lymphocyst formation, lymphedema). Infracolic omentectomy (total/partial) is recommended for stage I-II serous carcinoma, carcinosarcoma, and undifferentiated carcinoma (6). Aggressive histologies are consistently associated with higher recurrence rates (7, 8). Dilation and curettage, as a crucial preoperative assessment method for EC, can only obtain partial tumor tissue due to its blind procedure, resulting in merely 80% concordance with postoperative pathology (9–11). This limitation may compromise treatment decision-making accuracy, highlighting the persistent challenges in preoperative pathological evaluation. Conventional imaging modalities demonstrate limited capability in preoperative assessment of aggressiveness in EC. Therefore, it is particularly important to determine a method that can accurately predict the aggressiveness of EC prior to surgery.

Currently, artificial intelligence has been widely applied in the diagnosis, treatment, and prognosis detection of tumors (12, 13). Otani et al. built a multiparametric magnetic resonance images (MRI)-based machine learning classifier to predict histological grade in patients with EC, achieving an AUC of 0.77 (14). Xue et al. found that a combined model that incorporated clinical and radiomics features exhibited good performance in the prediction of histological grade in EC, achieving an AUC of 0.91 in the test set (15). While the results are promising, manual feature extraction remains time-consuming and lacks interpretability. To address these limitations, deep learning (DL) algorithms have been widely adopted in medical image analysis with growing clinical acceptance (16, 17). DL, as a branch of artificial intelligence, enables automatic feature extraction from medical images and demonstrates superior performance compared to manual feature extraction methods (18). The success of DL models relies heavily on large annotated datasets, yet the medical field faces significant challenges including limited annotation resources and data acquisition difficulties. Deep transfer learning (DTL) has thus emerged as a crucial solution to these obstacles (19, 20). DTL utilizes models pre-trained on large-scale natural image datasets (e.g., ImageNet) as foundational networks, leveraging their acquired knowledge to address medical image classification tasks. This approach significantly reduces the demand for extensive training data while simultaneously enhancing model convergence speed and classification performance (21). Dai et al. (22) found that a DL model based on non-enhanced Multiparametric MRI and clinical features can effectively differentiate uterine sarcomas from atypical leiomyomas, which is superior to radiomics. However, research on the non-invasive prediction of aggressiveness of EC using DTL model remains limited. Furthermore, fusion models demonstrate significantly superior generalization capability compared to individual machine learning models (23). Therefore, we incorporated corresponding algorithms to evaluate the overall predictive performance in this study.

In this study, we aim to develop DTL models, and clinical-DTL model for preoperatively predicting aggressive of EC based on multiparametric MRI before surgery, and to provide a basis for clinicians to make personalized and precise treatment plans.

## Materials and methods

### Patients

**Figure 1:**
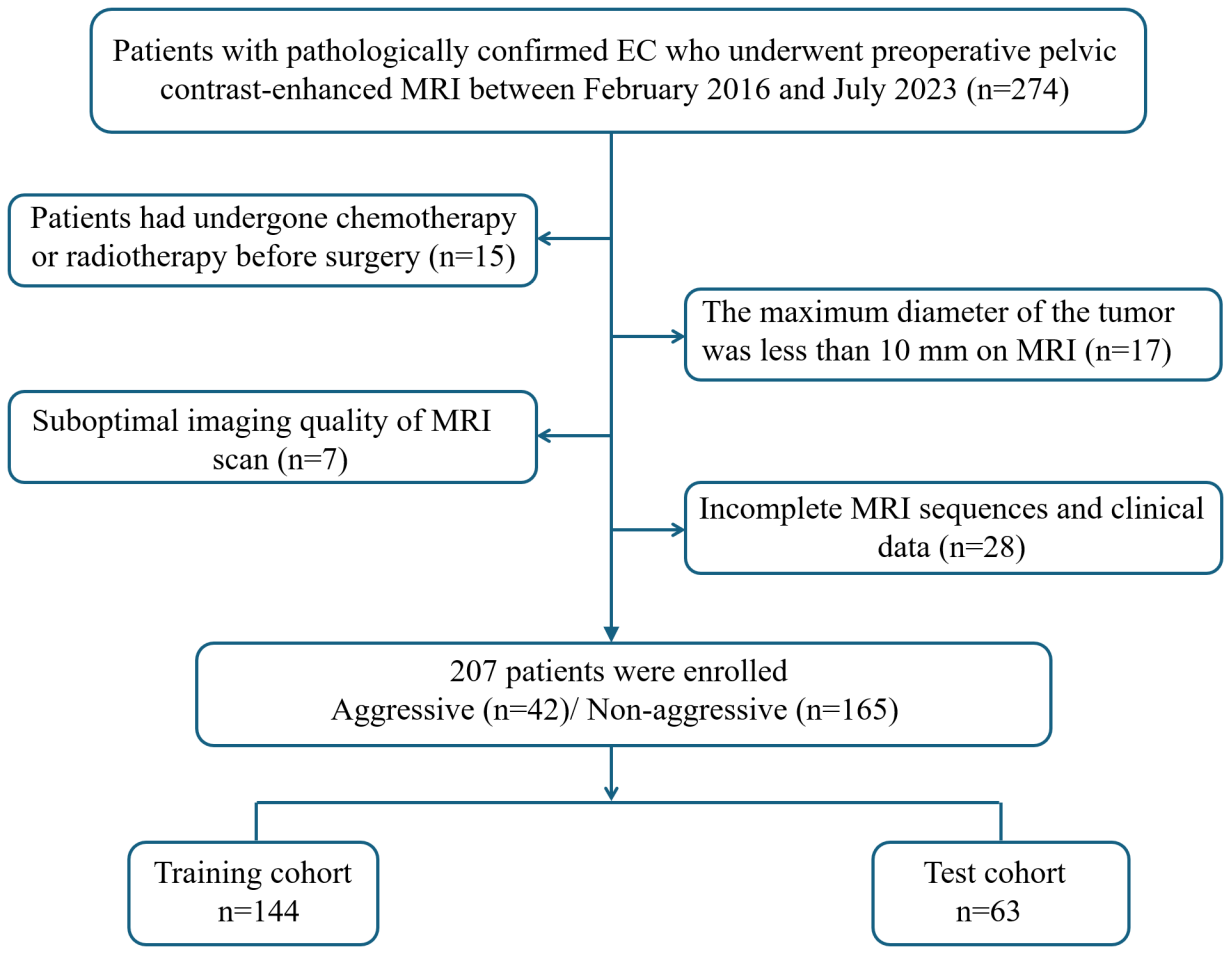
Flowchart of included and excluded patients with endometrial cancer.

This retrospective study was approved by the institutional review board of our center, and the requirement of informed consent was waived (Approval No: 2023-LHKY-086-01). A retrospective analysis was performed on patients who underwent continuous surgical resection of EC in our hospital from February 2016 to July 2023. The inclusion criteria were as follows (1): all patients underwent surgical treatment without preoperative chemoradiotherapy; (2) preoperative MRI performed within two weeks prior to surgery; (3) MRI sequences including T2-weighted imaging (T2WI), diffusion-weighted imaging (DWI), apparent diffusion coefficient (ADC) mapping, and contrast-enhanced T1-weighted imaging (CE-T1WI) sequences; and (4) complete clinical data. The exclusion criteria were as follows:(1) patients had undergone chemotherapy or radiotherapy before surgery; (2) the maximum diameter of the tumor was less than 10 mm on MRI; (3) suboptimal imaging quality of MRI scan; and (4) incomplete MRI sequences and clinical data. A flow chart of the inclusion and exclusion criteria for the patients is shown **Figure 1**. Clinical data were collected from all patients, including age, hypertension, diabetes, age at menarche, menopausal status, body mass index, CA125 and CA19–9 levels.

### Histologic grading and grouping

The histological grading of endometrioid carcinoma is primarily determined by the proportion of solid (non-glandular) growth: grade 1 tumors have ≤5% solid component, grade 2 have 6-50%, and grade 3 are defined by >50% solid growth (24). Aggressive histological types are composed of high-grade endometrioid carcinoma (grade 3), serous, clear cell, undifferentiated, mixed, mesonephric-like, gastrointestinal mucinous type carcinomas, and carcinosarcomas. Non-aggressive histological types are composed of low-grade (grade 1 and 2) endometrioid carcinoma (5).

### MR images acquisition and segmentation

MRI examination was performed using 3.0-T scanners (Siemens MAGNETOM Skyra and United Imaging uMR780) with an 8-channel coil. The imaging protocol included axial T2WI, DWI, ADC, and CE-T1WI. The detailed MRI parameters were as follows: Siemens MAGNETOM Skyra T2WI: Repetition time (TR)/Echo time(TE) 5,000 ms/77 ms, Field of view (FOV) 230 × 230 mm, matrix 320 × 320, slice thickness/gap 4 mm/1 mm; DWI: TR/TE 4,300 ms/57 ms, FOV 200 × 200 mm, matrix 160 × 160, slice thickness/gap 4 mm/4 mm; CE-T1WI: TR/TE 2.77ms/1.55 ms, FOV 340 × 340 mm, matrix 320 × 224, slice thickness/gap 3 mm/0 mm; United Imaging uMR780 T2WI: TR/TE 3,393 ms/81.6 ms, FOV 230 × 230 mm, matrix 320 × 320, slice thickness/gap 4 mm/1 mm; DWI: TR/TE 4,337 ms/78 ms, FOV 200 × 200 mm, matrix 160 × 160, slice thickness/gap 4 mm/4 mm; CE-T1WI: TR/TE 4.19 ms/1.99 ms, FOV 340 × 270 mm, matrix 320 × 224, slice thickness/gap 3 mm/0 mm. Axial CE-T1WI was performed by injecting gadobutrol (Gadavist; Bayer Healthcare Pharmaceuticals, Germany) at a rate of 2.0 mL/s and at a dosage of 0.2 mmol/L/kg.

The ITK-SNAP (version 3.8.0; https://www.itksnap.org) software was used to manually segment the lesion. A radiologist with 7 years of working experience manually delineated the region of interest (ROI) slice by slice, including cystic and necrotic areas, to generate a three-dimensional ROI volume of the tumor. After the ROI delineation was completed, another radiologist with 20 years of experience in imaging diagnosis reviewed the ROIs. In case of disagreement between the two, a third expert with 30 years of experience was consulted to provide a final decision.

### Preprocessing and DTL feature extraction

The patients were randomized (7:3) into a training cohort and a test cohort. For each patient, the slice displaying the largest ROI was chosen as the representative image. To streamline image complexity and mitigate background interference in the algorithmic model, only the minimal bounding rectangle of the ROI section was retained. Recent research on peritumoral regions informed our decision to expand this rectangle by an additional 10 pixels (25). To ensure uniform intensity distribution across RGB channels, Z-score normalization was applied to the images. The normalized images were then used as inputs for our model. We adopted real-time data augmentation techniques, such as random cropping and both horizontal and vertical flips, during the training process. For test images, however, only normalization was conducted.

### Development of DTL models

In this study, three classical network architectures-DenseNet121, ResNet101, and ResNet50-were employed. The neural networks were pre-trained on the ImageNet dataset, and partial pre-trained weights were transferred to the classification task in this study to facilitate knowledge transfer. Subsequently, a comprehensive fine-tuning strategy was adopted, where all network layers were set to an unfrozen state, enabling the model to deeply adapt to the current task and thereby enhance model performance. During training, data augmentation techniques such as random cropping were introduced to improve model robustness and generalization capability. Furthermore, the model was further optimized using a cosine decay learning rate scheduler, cross-entropy loss function, and stochastic gradient descent optimizer. A five-fold cross-validation strategy was applied to the training set to optimize and evaluate the final model. In the feature extraction stage, the penultimate layer of the fine-tuned convolutional neural network was utilized as a high-dimensional feature output layer.

The DenseNet121, ResNet101, and ResNet50 models were used to build DTL models based on single-sequence for T2WI, DWI, ADC, and CE-T1WI. To understand the distinct characteristics of each modality, we conducted a detailed comparative analysis of the results from each. Additionally, to explore the integration of multimodal and multi-model approaches, we combined their results using three different methods: mean, maximum, and minimum value fusion. The model with the best predictive performance was selected as the optimal DTL model in this study.

### Computational complexity analysis

The computational complexity of the models was systematically assessed using the following three standard metrics: 1) the number of parameters, indicating model size and memory footprint; 2) the number of floating-point operations per second (FLOPs), calculated for an input size of 224×224 to measure the theoretical computational cost; and 3) inference speed, reported in frames per second (FPS) and measured on an NVIDIA GeForce RTX 4090 GPU under FP16 precision with a batch size of 1 to simulate a realistic deployment scenario.

### Development of clinical model and clinical-DTLmodel

Univariate and multivariate logistic regression analyses were performed to identify clinical features associated with the aggressiveness in EC. Features demonstrating significant differences were considered independent risk factors and were subsequently used to establish clinical models using three distinct classifiers: ExtraTrees, Support Vector Machine, and RandomForest. The best-performing model was then chosen as the final clinical model. Furthermore, these significant clinical features were integrated with DTL predictions through logistic regression analysis to develop a combined clinical-DTL model, which was visualized using a nomogram. The final model integrated clinical and DTL features to improve prediction accuracy.

### Statistical analysis

Data analyses were performed using Python (version 3.7.12; https://www.python.org). Continuous variables are expressed as mean ± standard deviation, and categorical variables as frequencies (percentages). The Kolmogorov–Smirnov test was used to assess normality. Student’s t test or Mann-Whitney U test was used for continuous variables, and the Chi-square test or Fisher’s exact test was used for categorical variables. Variance inflation factor analysis was used to assess multicollinearity among the clinical variables. The area under the receiver operating characteristic curve (AUC), accuracy, sensitivity, and specificity were employed for the evaluation of each model. The optimal cutoff point for calculating accuracy, sensitivity, and specificity was determined by maximizing Youden’s index. Delong test was applied to compare the models. Calibration curves, Hosmer-Lemeshow analysis, and decision curve analysis were used to assess the performance of the models and net clinical benefit. *p* < 0.05 was considered statistically significant difference.

## Results

### Patients characteristics

A total of 207 patients were included. There were 144 patients in the training cohort (32 cases in aggressive and 112 cases in non-aggressive), 63 patients (10 cases in aggressive and 53 cases in non-aggressive) in the test cohort. According to the statistical results, none of the clinical characteristics were significantly different between the training and test cohorts (all *p*>0.05), and the clinical characteristics of the patients are listed in **Table 1**. Univariate and multivariate logistic regression analyses indicated that age and CA125 were independent risk factors for aggressive of EC (**Table 2**). To further evaluate the collinearity between age and CA-125, the variance inflation factors were calculated, which were 1.22 for age and 1.02 for CA-125, suggesting the absence of multicollinearity. The clinical model based on the ExtraTrees classifier demonstrated the highest AUC, with values of 0.830 (95% CI: 0.751–0.908) in the training cohort and 0.789 (95% CI: 0.643–0.934) in the test cohort. The AUCs achieved by the other two classifiers are presented in **Supplementary Table 1**.

**Table 1:** The clinical characteristics of the patients.

Clinical features	Training cohort (n=144)	Test cohort (n=63)	*p* value
Age (year)	60.02 ± 8.83	58.68 ± 8.79	0.316
Age_at_menarche (year)	14.97(14.00,16.00)	14.84 (13.00,16.00)	0.423
BMI (kg/m^2^)	26.41(22.50,30.72)	25.78 (22.00,29.15)	0.197
CA125 (U/ml)	45.19 (13.33,37.40)	59.51 (14.99,47.15)	0.176
CA19_9 (U/ml)	109.96 (9.48,36.78)	85.78 (9.99,38.68)	0.486
Menopause			0.374
no	25(17.36)	15(23.81)	
yes	119(82.64)	48(76.19)	
Hypertension			0.32
no	81(56.25)	30(47.62)	
yes	63(43.75)	33(52.38)	
Diabetes			0.972
no	98(68.06)	42(66.67)	
yes	46(31.94)	21(33.33)	

BMI, body mass index.

**Table 2:** Preoperative clinical risk factors for aggressive endometrial cancer.

Features	Univariate analysis		Multivariate analysis	
	OR (95%CI)	*p* value	OR (95%CI)	*p* value
Age	1.010 (1.003–1.016)	0.012	1.008 (1.002–1.014)	0.042
Age_at_menarche	0.986 (0.954–1.019)	0.483		
BMI	0.997 (0.986–1.008)	0.658		
CA125	1.002 (1.001–1.002)	0.000	1.002 (1.001–1.002)	<0.001
CA19_9	1.001 (0.999–1.003)	0.211		
Hypertension	1.000 (0.890–1.123)	1.000		
Menopause	1.078 (0.926–1.255)	0.414		
Diabetes	1.093 (0.966–1.236)	0.235		

OR, odds ratio; CI, confidence interval.

### The performance of the DTL models based on single-MRI sequence

**Figure 2:**
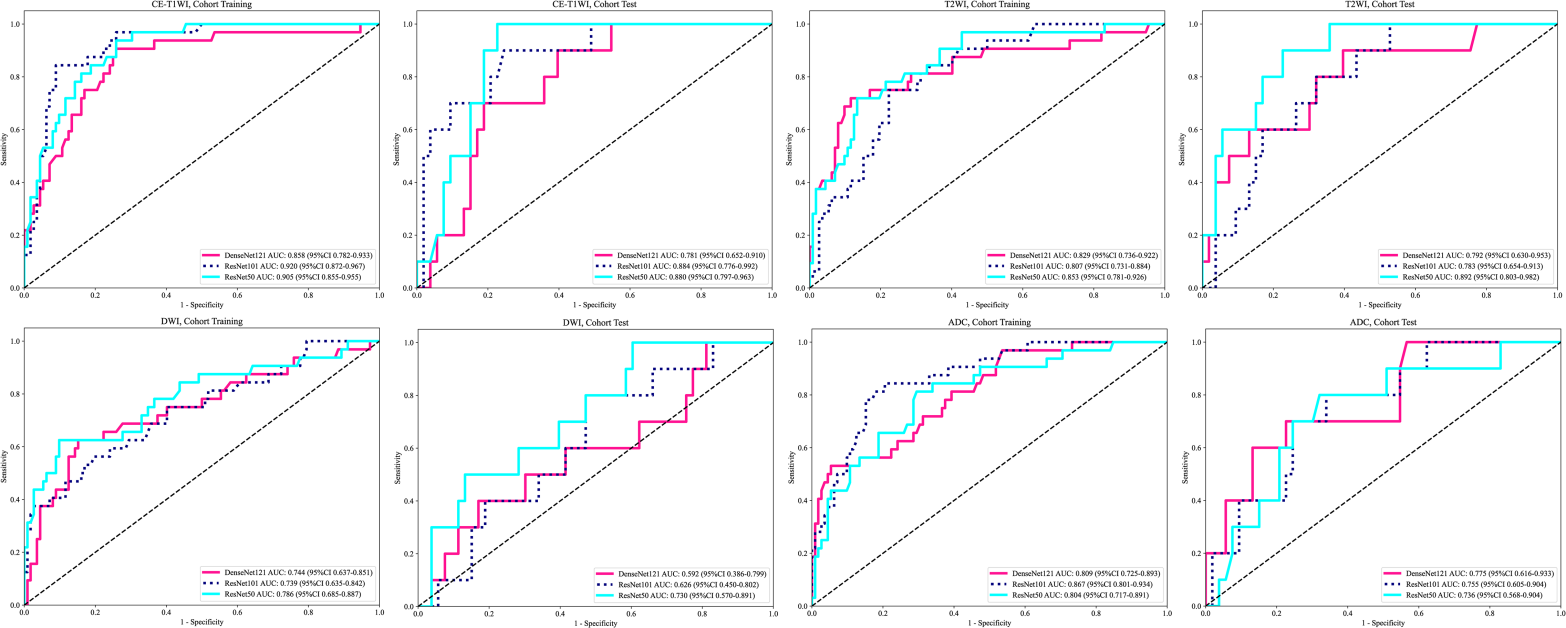
Receiver operating characteristic curves of the different deep transfer learning models.

The performance of the DTL models based on singe-MRI sequence is shown in **Table 3**. The ROC curves of the different DTL models are shown in **Figure 2**. In the training cohorts, all twelve DTL models demonstrated satisfactory diagnostic performance in predicting EC aggressiveness, with AUC values ranging from 0.744 (95% CI: 0.637–0.852) to 0.920 (95% CI: 0.872–0.967). In the test cohorts, the model based on DWI showed relatively low diagnostic performance, while the remaining nine models achieved AUC values between 0.736 (95% CI: 0.568–0.904) and 0.892 (95% CI: 0.803–0.982). Among them, the ResNet101 model based on CE-T1WI performed best, with AUCs of 0.920 (95% CI: 0.872–0.967) in the training cohort and 0.884 (95% CI: 0.776–0.992) in the test cohort. This was followed by the ResNet50 model, which achieved AUCs of 0.905 (95% CI: 0.855–0.955) in the training cohort and 0.880 (95% CI: 0.780–0.963) in the test cohort. Among the models based on T2WI, ResNet50 exhibited the highest performance, with an AUC of 0.853 (95% CI: 0.781–0.926) in the training cohort and 0.892 (95% CI: 0.803–0.982) in the test cohort.

**Table 3:** The performance of the deep transfer learning models based on single-MRI sequence.

Sequences	Models	AUC (95% CI)	Accuracy	Sensitivity	Specificity
CE-T1WI					
Training	DenseNet121	0.858 (0.782–0.933)	0.771	0.875	0.741
	ResNet101	0.920 (0.872–0.967)	0.889	0.812	0.911
	ResNet50	0.905 (0.855–0.955)	0.778	0.906	0.741
Test	DenseNet121	0.781 (0.652–0.910)	0.778	0.600	0.811
	ResNet101	0.884 (0.776–0.992)	0.778	0.800	0.774
	ResNet50	0.880 (0.797–0.963)	0.794	0.900	0.774
T2WI					
Training	DenseNet121	0.829 (0.736–0.922)	0.840	0.687	0.884
	ResNet101	0.807 (0.731–0.884)	0.764	0.719	0.777
	ResNet50	0.853 (0.781–0.926)	0.826	0.687	0.866
Test	DenseNet121	0.792 (0.630–0.953)	0.635	0.800	0.604
	ResNet101	0.783 (0.654–0.913)	0.683	0.700	0.679
	ResNet50	0.892 (0.803–0.982)	0.778	0.800	0.774
DWI					
Training	DenseNet121	0.744 (0.637–0.852)	0.792	0.594	0.848
	ResNet101	0.739 (0.635–0.842)	0.750	0.531	0.812
	ResNet50	0.786 (0.685–0.887)	0.833	0.594	0.902
Test	DenseNet121	0.592 (0.386–0.799)	0.746	0.300	0.830
	ResNet101	0.626 (0.450–0.802)	0.556	0.700	0.528
	ResNet50	0.730 (0.570–0.891)	0.476	0.900	0.396
ADC					
Training	DenseNet121	0.809 (0.725–0.893)	0.847	0.500	0.946
	ResNet101	0.867 (0.801–0.934)	0.799	0.812	0.795
	ResNet50	0.804 (0.717–0.891)	0.729	0.781	0.714
Test	DenseNet121	0.775 (0.616–0.933)	0.746	0.600	0.774
	ResNet101	0.755 (0.605–0.904)	0.667	0.700	0.660
	ResNet50	0.736 (0.568–0.904)	0.698	0.700	0.698

CE-T1WI, contrast-enhanced T1-weighted imaging; T2WI, T2 weighted imaging; DWI, diffusion weighted imaging; ADC, apparent diffusion coefficient; AUC, the area under the receiver operating characteristic curve; CI, confidence interval.

### Model interpretation

**Figure 3:**
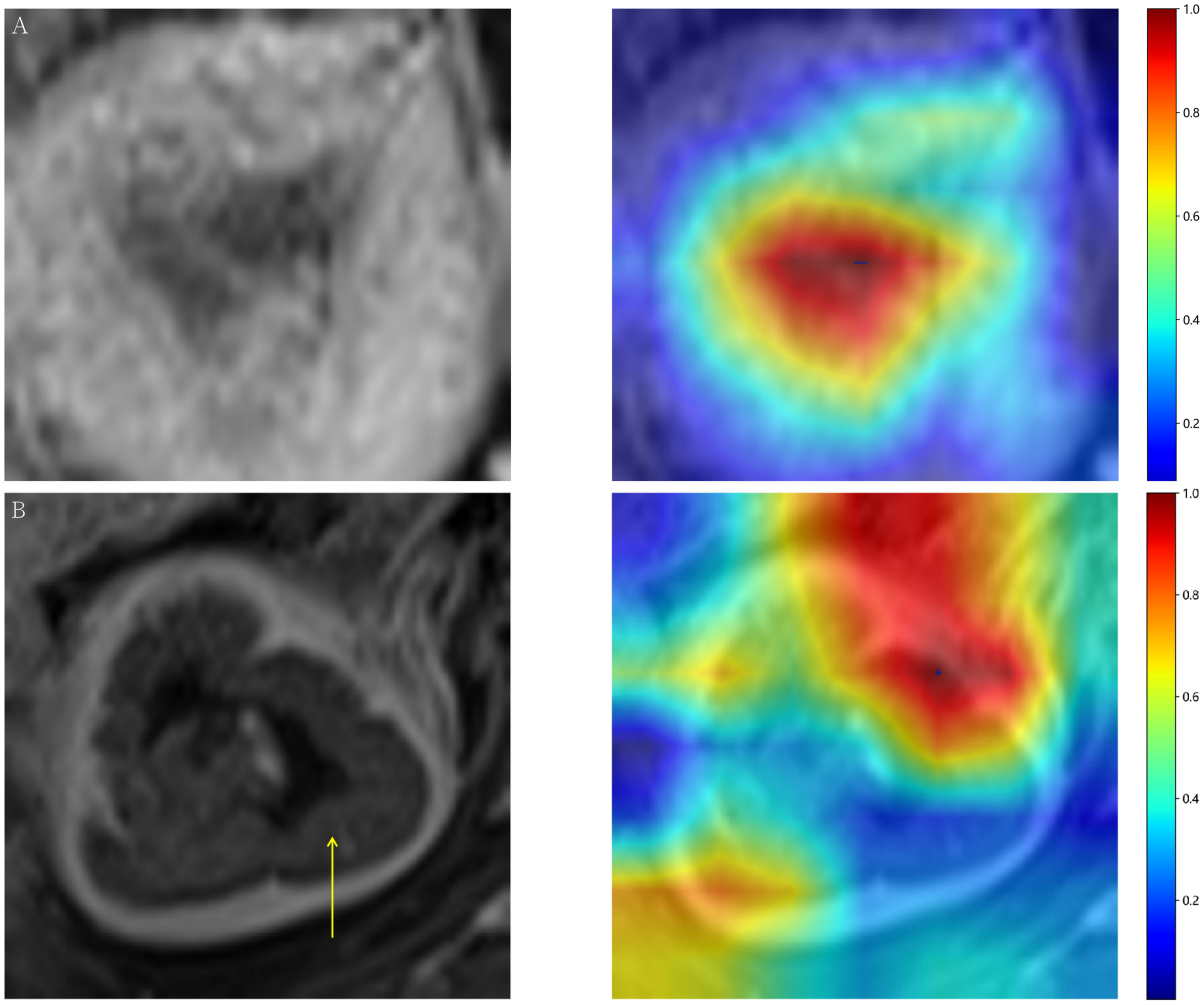
**(A)** Representative heatmap of a successful prediction in the test dataset, constructed using the Gradient-weighted Class Activation Mapping (Grad-CAM) technique. **(B)** Prediction failure visualized with Grad-CAM, showing an example where a tumor image was incorrectly classified as negative.

Gradient-weighted Class Activation Mapping (Grad-CAM) visually illustrates the decision-making basis of models through heatmaps, where varying color intensities represent the attention strength of convolutional neural networks to different image regions. In our study, the CE-T1WI modality demonstrated the most consistent model performance across all tested modalities. **Figure 3** exemplarily presents the application of Grad-CAM by visualizing activated regions in the final convolutional layer for cancer type prediction. **Figure 3A** presents heatmap generated by the Grad-CAM technique, displaying tumor probability estimate and the corresponding inputted MRI image. The region highlighted by the DTL model show strong concordance with area that radiologist identified as critical for EC assessment. **Figure 3B** illustrates a representative misclassification case from the test dataset, where a tumor was incorrectly predicted as negative, with the yellow arrow pointing to the tumor location. This visualization technique helps identify which image areas exert the most critical influence on model predictions, thereby providing essential insights for understanding the decision-making process and enhancing model interpretability.

### The optimal DTL model

The performance of the different fusion models for predicting aggressive is shown in **Table 4**. The mean fusion model was selected as the optimal prediction model with the highest AUC of 0.925(95%CI: 0.859–0.990) in the test cohort. Notably, due to the suboptimal performance of the DWI model on the test cohort, we excluded the DWI modality from our fusion process. However, for comprehensive insights, the results including the DWI modality can be found in **Supplementary Table 2**. A systematic comparison of the computational complexity of each model was conducted to assess the practical utility and deployment potential of the proposed hybrid model, as shown in **Table 5**.

**Table 4:** The performance of different fusion model.

Cohorts	Models	AUC (95% CI)	Accuracy	Sensitivity	Specificity
Training	mean	0.963 (0.933–0.992)	0.931	0.844	0.955
	maximum	0.930 (0.889–0.971)	0.799	0.937	0.759
	minimum	0.922 (0.865–0.980)	0.840	0.875	0.830
Test	mean	0.925 (0.859–0.990)	0.810	0.900	0.792
	maximum	0.861 (0.770–0.952)	0.746	0.900	0.717
	minimum	0.905 (0.820–0.990)	0.873	0.700	0.906

AUC, the area under the receiver operating characteristic curve; CI, confidence interval.

**Table 5:** Comparison of computational complexity (Input size: 3×224×224).

Model	Params (M)	FLOPs (G)	Inference Speed (FPS)
ResNet50	25.5	4.1	~2050
ResNet101	44.5	7.8	~1250
DenseNet121	7.9	2.9	~2450

FLOPs, Floating-point operations per second; FPS, Frames Per Second.

### Clinical and combined model

**Figure 4:**
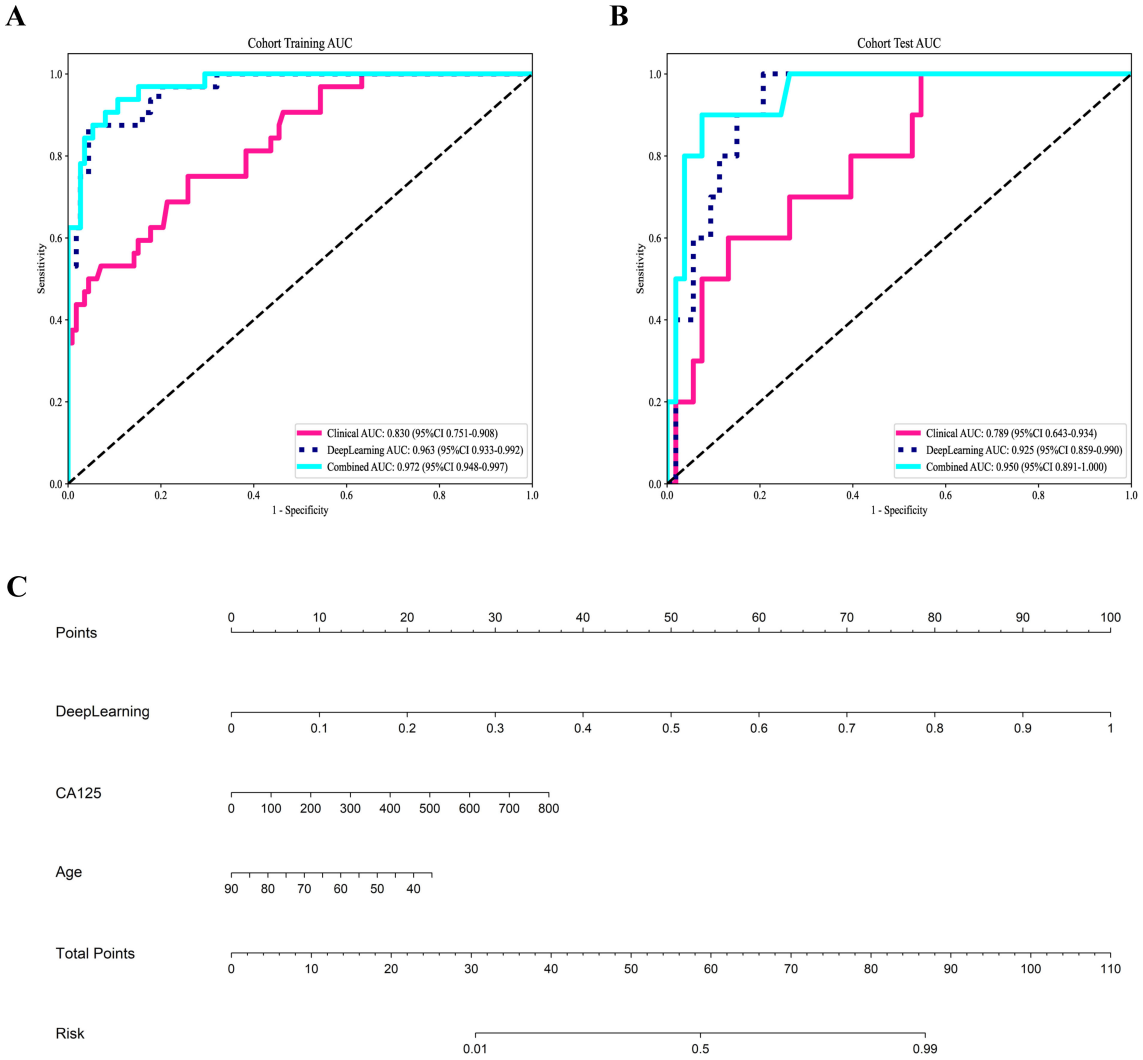
Receiver operating characteristic curves of the three models for predicting aggressive endometrial cancer in the training **(A)** and test cohort **(B)**; **(C)** Nomogram for the prediction of aggressive endometrial cancer.

A combined model based on DTL and clinical features was established, and visualized by a nomogram. The diagnostic performance of the combined model for aggressive EC in the two cohorts is presented in **Table 6**. The ROC curves of the different models are shown in **Figures 4A, B**. The combined model demonstrated the best diagnostic performance, achieving AUC of 0.967 (95% CI: 0.948–0.997) and 0.950 (95% CI: 0.891–1.000) in the training and test cohorts, respectively. These results were significantly superior to those of the clinical model (*p* < 0.001 and 0.021, respectively), but showed no statistically significant difference compared to the DTL model. Comparisons of the AUCs among the other models are provided in **Supplementary Figure 1**. For the combined model, the areas under the precision-recall curve were 0.925 (95% CI: 0.882-0.968) and 0.782 (95% CI: 0.680-0.884) for the training and test cohorts, respectively.

**Table 6:** Diagnostic performance of the clinical, deep transfer learning, and combined model for aggressive of endometrial cancer.

Cohorts	Models	AUC (95% CI)	Accuracy	Sensitivity	Specificity
Training	Clinical	0.830 (0.751– 0.908)	0.743	0.750	0.741
	DTL	0.963 (0.933– 0.992)	0.938	0.875	0.955
	Combined	0.972 (0.948– 0.997)	0.903	0.937	0.893
Test	Clinical	0.789 (0.643 – 0.934)	0.825	0.600	0.868
	DTL	0.925 (0.859– 0.990)	0.825	1.000	0.792
	Combined	0.950 (0.891– 1.000)	0.921	0.900	0.925

DTL, deep transfer learning.

**Figure 5:**
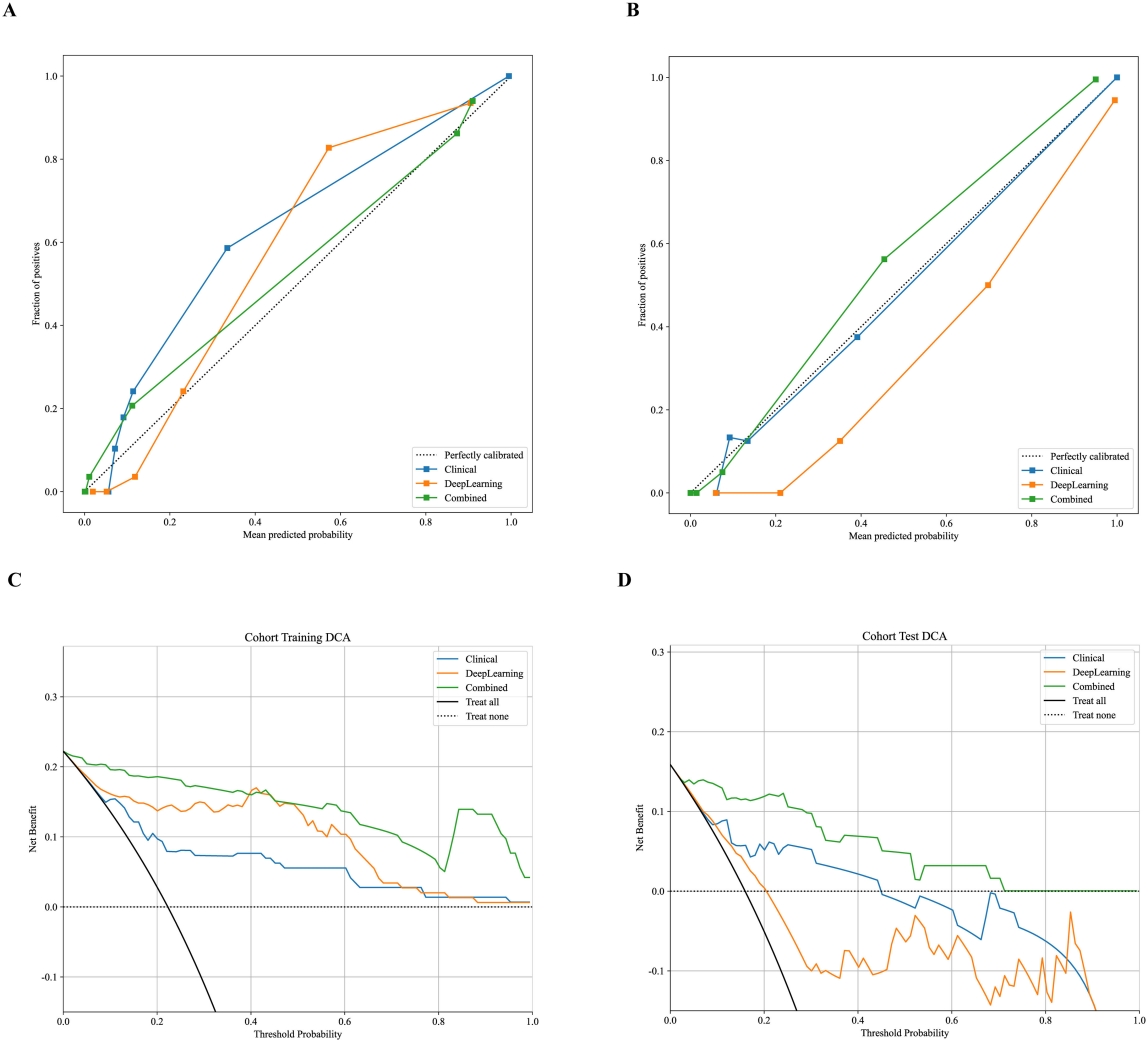
Calibration curves for predicting aggressive endometrial cancer probability in the training **(A)** and test cohort **(B)**. Decision curve analysis of all models for patients in the training **(C)** and test cohort **(D)**.

**Figure 4C** shows the nomogram. The nomogram intuitively predicted the risk of aggressiveness in EC, with the final formula as follows: 16.3354 * DL + 0.0074 * CA125 + -0.0677 * Age -3.4869056170053083. The calibration curve indicated good agreement between the predicted values of the combined model and the actual observations, with Brier scores of 0.056 and 0.075 in the training and test cohorts, respectively. The Hosmer-Lemeshow test results in both the training and test cohorts demonstrated adequate model fit (*p* = 0.890 and 0.445, respectively) (**Figures 5A, B**). Decision curve analysis further revealed that the combined model provided higher net benefit across a wide threshold probability range in both the training (approximately 1% to 100%) and test (approximately 2% to 70%) cohorts (**Figures 5C, D**), demonstrating broader clinical utility.

## Discussion

In this study, a combined model based on preoperative multiparametric MRI-derived DTL features and incorporating clinical features achieved the best performance for classifying EC aggressiveness. The non-invasive prediction model constructed in this study may provide clinicians with new therapeutic guidance for treating EC patients.

Early-stage nonaggressive EC demonstrates favorable prognosis with minimal recurrence risk, in contrast to advanced-stage aggressive variants which exhibit poor clinical outcomes (26). Akçay A et al. found that ADC measurements can helps to differentiate histological grade (27). However, Bonatti M et al. reported that ADC values didn’t show any statistically significant correlation with tumor grade (28). In addition to conventional imaging methods, Yue et al. found that diffusion kurtosis imaging could be used for histological grade assessments of EC (29). Unlike conventional MRI sequences, advanced DWI techniques require higher main magnetic field strength and gradient performance, which may limit their clinical utility. Since these functional imaging sequences are not part of standard protocols, they prolong examination time and increase the risk of scan failure. Two studies developed combined models based on radiomics features and clinical features of patients to predict the pathological grade of EC (30, 31). However, due to the manual feature extraction required by radiomics, as well as the redundancy of the extracted features, further studies are needed.

DTL has been successfully applied to automatic segmentation of EC lesions, assessment of myometrial infiltration, and differentiation of benign and malignant lesions (32–35). The features extracted by DTL are automatically learned from the data, avoiding manual feature extraction, and are usually more competitive in classification performance than traditional radiomics methods. In this study, we leveraged three deep neural network architectures (ResNet50, ResNet101, and DenseNet121), pre-trained on ImageNet. These models were subsequently fine-tuned to predict the aggressiveness of EC. Given the complementary value of different MRI sequences in characterizing tumor features, we developed DTL models incorporating features extracted from multiple MRI sequences to identify the optimal approach for predicting tumor differentiation of EC. Our results demonstrated that the DTL model based on CE-T1WI sequences exhibited superior predictive capability, showing the most consistent performance across all three CNN architectures. This finding aligns with previous studies on nasopharyngeal cancer and cervical cancer (36, 37). This may be attributed to the high malignancy and vascular density of aggressive EC, which creates distinct contrast uptake patterns after administration and enhances heterogeneity between aggressive and non-aggressive.

Recent studies suggest that decision-level fusion (late fusion), which integrates predictions from multiple models, outperforms both feature-level fusion (early fusion) and single-model approaches in diagnostic performance (23). In this study, maximum, minimum, and mean fusion strategies were applied to integrate multi-modal outcomes at the decision level, leading to further improvement in model performance. The average fusion method achieved higher AUC values of 0.925 in the test cohort. This finding is consistent with Ueno et al. (38), who also demonstrated that combining texture features from multiple sequences provides better predictive performance than single-sequence features. A combined model incorporating DTL and clinical features achieved an AUC of 0.950 in the test set, with specificity increasing from 0.792 to 0.925. This indicates that clinical variables provide significant value in enhancing model specificity. Although the overall performance improvement was modest, potentially due to the limited incremental information contributed by the clinical model, these findings still establish an important foundation for developing more precise multimodal predictive frameworks. Future studies could focus on integrating advanced fusion architectures such as attention mechanisms, or incorporating additional high-value clinical variables to further optimize model performance.

Due to the fact that ResNet can capture more feature information, has stronger stability, and better model accuracy and generalization ability (39), we chose ResNet and compared two commonly used ResNet models: ResNet50 and ResNet101. From the perspective of computational complexity analysis, the FLOPs of ResNet101 (7.8G) are 1.9 times those of ResNet50 (4.1G), while its AUC value on the CE-T1WI test cohort (0.884) shows an 11.3% improvement over ResNet50 (0.794). This indicates that although increasing network depth significantly raises computational cost, it may also lead to optimized classification performance by enabling the learning of more complex hierarchical feature representations. Compared to ResNet, DenseNet constructs a model through dense connections, with each layer’s input including the outputs of all previous layers, improving information transmission efficiency (40). Theoretically, the performance is better than ResNet. However, ResNet101 achieves a performance improvement through a moderate increase in computational cost, indicating that based on specific clinical analysis, ResNet may be more suitable for distinguishing the types of EC.

The 2023 FIGO staging system elevates histological type to a central role in risk stratification: aggressive histological types with any myometrial involvement are classified as stage IIC, while those without myometrial involvement are classified as stage IC. These categories differ significantly in both treatment strategies and prognosis, with extensive database series and retrospective reports consistently demonstrating higher recurrence rates in aggressive histological types (41, 42). Thus, the DTL model developed in our study for noninvasive and accurate preoperative prediction of histological subtypes carries important clinical implications. The model not only substantially advances the decision-making timeline—providing critical guidance to clinicians before postoperative pathology reports become available, thereby enabling personalized surgical planning—but also creates valuable opportunities for early adjuvant therapy strategizing. If integrated into clinical systems, this tool could help mitigate biases arising from diagnostic delays or inter-observer variability among pathologists, effectively preventing both overtreatment and undertreatment of patients.

This study has several limitations. First, as a single-center retrospective investigation, the fusion model was selected based on its performance on the hold-out test set, which may lead to performance overestimation, prospective multi-center validation is necessary prior to its clinical application. Second, although the sample size was relatively limited, the use of transfer learning still contributed to effective predictive performance. Third, we only selected the largest cross-sectional image of the tumor for DTL feature extraction, which may lose some important three-dimensional information inside the EC. Fourth, potential bias from scanner heterogeneity may affect the model’s generalizability. Finally, manual segmentation of individual cases is highly time-consuming and, to some extent, lacks standardization. Subsequent studies will employ DL techniques to automatically segment the entire tumor, which will help reduce operator-dependent variability and enhance workflow efficiency.

Our combined model, which incorporates DTL and clinical features based on multiparametric MRI, has the potential to distinguish aggressive EC. This capability can aid clinicians in tailoring individualized treatment strategies for EC patients.

## Data Availability

The raw data supporting the conclusions of this article will be made available by the authors, without undue reservation.
