# Supplementary material for: Preoperative prediction of aggressive endometrial cancer using multiparametric MRI-based deep transfer learning models
Source: Front Oncol. 2025 Nov 18;15:1694223. doi: 10.3389/fonc.2025.1694223 (PMC12668923; doi:10.3389/fonc.2025.1694223)
Supplement: Supplementary file 1 [file Table1.docx]

**Supplementary Material**

We employed three machine learning algorithms to model the features identified in univariate analysis. It was observed that non-linear models outperformed linear models in terms of model performance. For subsequent comparisons, we selected the Extratrees model as the final clinical signature.

Supplementary Table S1 Diagnostic performance of the clinical models for different classifiers.

| Cohorts | Models | AUC (95% CI) | | Accuracy | Sensitivity | Specificity |
| --- | --- | --- | --- | --- | --- | --- |
| Training | ExtraTrees | | 0.830 (0.751– 0.908) | 0.833 | 0.250 | 1.000 |
|  | RandomForest | | 0.820 (0.740 - 0.901) | 0.854 | 0.375 | 0.991 |
|  | SVM | | 0.708 (0.601 - 0.814) | 0.826 | 0.281 | 0.982 |
| Test | ExtraTrees | | 0.789 (0.643 - 0.934) | 0.841 | 0.300 | 0.943 |
|  | RandomForest | | 0.772 (0.617 - 0.926) | 0.841 | 0.400 | 0.925 |
|  | SVM | | 0.706 (0.482 - 0.929) | 0.841 | 0.400 | 0.925 |

SVM: Support Vector Machine; AUC: the area under the receiver operating characteristic curve; CI: confidence interval

**Fusion Results with DWI**

The fusion results incorporating DWI modality, specifically using mean and maximum fusion methods, showed a decline in performance compared to the results without adding DWI. This suggests that the inclusion of DWI may not enhance and may even slightly hinder the overall efficacy of the fusion methods in this context. The performance of the models is shown in Supplementary Table S2.

Supplementary Table S2 Metric of different fusion methods.

| Cohorts | Models | AUC (95% CI) | Accuracy | Sensitivity | Specificity |
| --- | --- | --- | --- | --- | --- |
| Training | mean | 0.968 (0.943–0.994) | 0.910 | 0.875 | 0.920 |
|  | maximum | 0.928 (0.884–0.971) | 0.778 | 0.937 | 0.732 |
|  | minimum | 0.912 (0.854–0.970) | 0.826 | 0.875 | 0.812 |
| Test | mean | 0.913 (0.841–0.985) | 0.857 | 0.800 | 0.868 |
|  | maximum | 0.829 (0.724–0.935) | 0.683 | 0.900 | 0.642 |
|  | minimum | 0.903 (0.824–0.981) | 0.794 | 0.900 | 0.774 |

AUC, The area under the receiver operating characteristic curve; CI, Confidence interval
