# Supplementary figures and images for: Preoperative prediction of aggressive endometrial cancer using multiparametric MRI-based deep transfer learning models
Source: Front Oncol. 2025 Nov 18;15:1694223. doi: 10.3389/fonc.2025.1694223 (PMC12668923; doi:10.3389/fonc.2025.1694223)

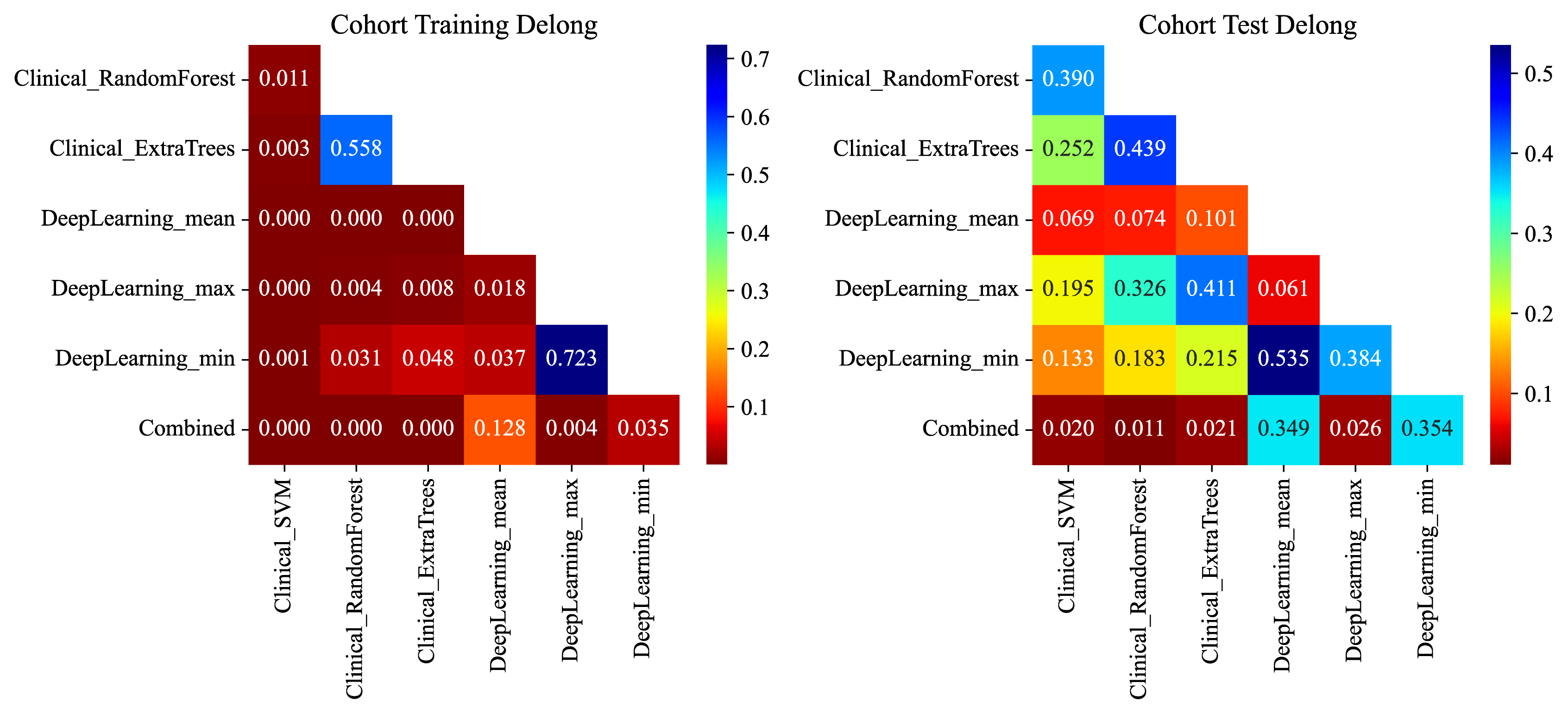

Supplement: Supplementary file 2 [file Image1.tif]
